# An online exploratory survey of Australian athletes’ and exercisers’ use of and attitudes towards ultra-processed sports foods

**DOI:** 10.1017/S0007114523000648

**Published:** 2023-11-14

**Authors:** Adrienne Forsyth, Evangeline Mantzioris

**Affiliations:** 1 School of Allied Health, Human Services and Sport, La Trobe University, Melbourne, Australia; 2 School of Behavioural and Health Sciences, Australian Catholic University, Melbourne, Australia; 3 Clinical and Health Sciences & Alliance for Research in Exercise, Nutrition and Activity (ARENA), University of South Australia, Adelaide, Australia

**Keywords:** Athletes, Exercisers, Sports foods, Ultra-processed foods, Attitudes

## Abstract

Sports foods are convenient alternatives to everyday foods to fuel performance. Strong scientific evidence supports their use; however, commercial sports foods are classified by the NOVA system as ultra-processed foods (UPF). Consumption of UPF has been associated with poor mental and physical health, but little is known about athletes’ consumption of and attitudes towards sports foods as a source of UPF. The aim of this cross-sectional study was to assess Australian athletes’ intake of and attitudes towards sports foods and UPF. Adult athletes were recruited to complete an anonymous online survey via social media between October 2021 and February 2022. Data were analysed using descriptive statistics, and Pearson’s *χ* ^2^ test was used to assess potential relationships between categorical demographic variables and consumption of sports foods. One hundred forty Australian adults participating in recreational (*n* 55), local/regional (*n* 52), state (*n* 11), national (*n* 14) or international (*n* 9) sports completed the survey. Ninety-five percent reported consuming sports foods within the past 12 months. Participants consumed sports drinks most commonly (73 %) and isolated protein supplements most frequently (40 % at least once per week). Participants reported everyday foods to be more affordable, taste better, present less risk of banned substances, but less convenient and greater risk of spoilage. Half (51 %) of participants reported concern about health effects of UPF. Participants reported regular UPF consumption despite taste and cost-related preferences for everyday foods and health concerns regarding UPF intake. Athletes may need support to identify and access safe, affordable, convenient, minimally processed alternatives to sports foods.

The NOVA system is the most widely accepted method of classifying food based on the nature, extent and purpose of food processing^(1,2)^. According to the NOVA classification system, ultra-processed foods (UPF) are not simply modified foods. Rather, they are formulations made using ingredients not typically found in a household pantry such as industrial food substances and additives^(1)^. UPF are often attractively packaged and extensively marketed as convenient replacements for less processed or whole foods^(3)^. Typically high in saturated fat, sugar and salt and low in micronutrients and fibre, UPF are often, but not always, energy dense and nutrient poor^(1)^. They are not recommended in national dietary guidelines, with consumption actively discouraged by some (i.e. Brazil^(4)^). However, recent evidence has shown high consumption across Western countries of up to 60 % energy intake. Emerging evidence indicates that UPF consumption is associated with poor mental and physical health, including CVD, cerebrovascular disease, depression and all-cause mortality^(5–10)^. These risks remain when data are adjusted for diet quality or pattern, suggesting that UPF intake increases the risk of chronic disease regardless of an otherwise healthy diet^(11)^.

Sports nutrition guidelines recommend that athletes ‘fuel for the work required’^(12,13)^, with periodised nutrition plans individually tailored for athletes’ training, competition and physique goals^(14)^. Fuelling recommendations use a food-first approach, recognising that a carefully planned whole-food diet provides appropriate fuel and hydration for performance and recovery^(15,16)^. Additionally, whole foods provide athletes with all nutrients and bioactive compounds needed to promote and support health^(17–19)^.

Sports foods are a type of sports supplement, defined as ‘specialised products used to provide a convenient source of nutrients when it is impractical to consume everyday foods’^(20,21)^. They contain nutrients tailored to support exercise-related goals and packaged conveniently as sports drinks, gels, confectionery, electrolyte supplements, isolated protein supplements, and mixed macronutrient supplements such as sports bars, powders, and liquid meals (see Table 1). Sports foods are effective in specific situations to achieve hydration, fuelling, recovery, training adaptation and electrolyte balance^(22,23)^. They also provide safe alternatives where there are food intolerances, allergies, preferences, limited energy budgets, limited availability of foods, food hygiene or contamination risks^(17,24)^. However, sports foods are intended for sport-specific use, not to replace an everyday diet. They do not provide all the nutrition needed for good health^(22)^. Furthermore, due to the nature of their production and formulation, commercial sports foods are typically classified according to the NOVA system as UPF.

**Table 1.** Sports foods included in the Australian Sports Supplement Framework^(31)^

Sports food	Description
Sports drinks	Carbohydrate-electrolyte drinks
Sports gels	Concentrated source of carbohydrate of honey consistency
Sports confectionery	Concentrated source of carbohydrate in chewy candy form
Sports bars	Compact source of carbohydrate in bar form
Electrolyte supplement	Electrolyte replacement in powder, tablet or ready-to-drink form
Isolated protein supplement	High-protein (> 90 %) powders, bars or drinks
Mixed macronutrient supplement (bar, powder, liquid meal)	Compact source of varied amounts of macronutrients including carbohydrate and protein, and may also contain micronutrients and other performance-enhancing ingredients

Sports nutrition recommendations and studies of dietary intake in athletes tend to focus on energy, macronutrients and select micronutrients as they relate to performance^(15,25)^. Less is understood about dietary patterns and diet quality in athletes, and few studies have focused on athletes’ intake of sports foods. Those that have assessed sports food intake have predominantly done so as part of a broad investigation of sports supplement use. Garthe and Maughan^(21)^ reviewed the published literature in 2018, mostly of studies now more than 10 years old, and found supplement use by 40–100 % of athletes. They cited sports supplements, vitamin and mineral supplements, and herbs as the most used supplements. It is unclear what proportion of these sports supplements were sports foods. Reasons cited for consuming sports supplements were direct performance benefits, health benefits, following advice or practice of esteemed peers or coaches, a ‘just in case’ approach, convenience or financial sponsorship^(21)^. A review of dietary supplement use (including sports foods) by masters athletes found that approximately 60 % of masters athletes used supplements, predominantly for health reasons^(26)^. Other recent studies have found high rates (49–97 %) of sports supplement use among young elite athletes^(27–30)^. While evidence remains limited, Maughan *et al.* ^(23)^ concluded that athlete supplement use varies according to sports/activities, is greater in men, is influenced by others and increases with age and level of training/performance.

The evidence to date suggests many athletes consume sports supplements including sports foods. As commercial sports foods are UPF, and UPF consumption is associated with nutrition-related chronic disease, it is timely to consider the impacts of UPF consumption for athletes. National sporting authorities provide sports supplements and sports foods guidelines^(31)^. However, there are no current recommendations for athletes regarding UPF consumption, and no studies to our knowledge investigating the intake of sports foods as a source of UPF in athletes. To support the initiation of research in this space, the aim of this study was to assess athletes’ and exercisers’ intake of and attitudes towards ultra-processed sports foods. This research will identify whether sports foods, as a source of UPF, are commonly consumed by athletes and whether athletes and exercisers are concerned about UPF consumption. This knowledge will inform future interventions and research to promote nutrition-related health for athletes.

## Materials and methods

### Study design

We used an anonymous, online, cross-sectional survey to assess athletes’ and exercisers’ intake of and attitudes towards sports foods.

### Participants and setting

We recruited a convenience sample of athletes and exercisers via social media (Twitter, LinkedIn, Facebook) between October 2021 and February 2022. Eligible participants were Australian residents, aged 18+ years, able to read and write in English, and met minimum physical activity criteria (participate in recreational or competitive sport or engage in regular physical training defined as at least three sessions of moderate or vigorous physical activity per week over the past month^(32,33)^). Participants were limited to Australian residents because the survey used language aligned with the Australian Institute of Sport Sports Supplement Framework^(31)^ and commercial Australian sports foods.

### Survey tool

The survey was adapted from a previously validated survey designed to assess intake and motivations for intake of dietary supplements^(34)^ and administered anonymously online via QuestionPro. Multi-select and free-text questions were used to ask about demographic characteristics (age, gender, income); level, type and frequency of sport participation; use of special diets; frequency of consumption of sports foods over the past 12 months; reasons for choosing or not choosing to consume sports foods; everyday foods used in place of sports foods; and whether participants were concerned about UPF consumption (see Table 2). The adapted survey was developed by academic sports dietitians and piloted for comprehension and face validity with four practising sports dietitians and four athletes competing in state or national level sports. Updates to the survey based on their feedback included a section identifying reasons for not consuming sports foods for non-consumers and ‘accommodate reduced/increased energy budgets or macronutrient targets’ as a reason for consuming or not consuming sports foods.

**Table 2.** Survey questions

Question	Response options
What is your age?	• Under 18 years • 18–24 years • 25–34 years • 35–44 years • 45–54 years • 55–64 years • 65–75 years • Greater than 75 years
What is your gender?	• Woman • Man • Non-binary • Prefer not to say • Prefer to self-describe:
Do you live in Australia?	• Yes • No
What is your residential postcode?	(Free-text)
What range most closely represents your total annual household income?	• $0–$24 999 • $25 000–$49 999 • $50 000–$74 999 • $75 000–$99 999 • $100 000–$124 999 • $125 000–$149 999 • $150 000–$174 999 • $175 000 - $199 999 • $200 000 or greater • Prefer not to disclose
What sort of sport or exercise do you participate in on a regular basis?	• Team sport • Individual sport • Non-competitive exercise • Other
What level of sport do you participate in on a regular basis?	• Recreational • Local or regional competition • State competition • National competition • International competition • Other • N/A
What is the main sport that you play? If you do not play sport, what is the main type of exercise that you do?	(Free-text)
How many hours do you spend exercising, training, or competing each week?	• 0–4 h • 5–9 h • 10–14 h • 15–19 h • 20 or more hours
Do you follow any special diets? Please select all that apply	• No special diet • Flexitarian • Vegetarian • Vegan • Low fat • Low carbohydrate • Ketogenic • Gluten free • Low FODMAP • Other
In the past 12 months, how often have you consumed each of the following sports foods? • Sports gels • Sports confectionery • Sports bars • Sports drinks • Electrolyte supplement • Isolated protein supplement • Mixed macronutrient supplement (liquid meals) • Mixed macronutrient supplement (powders) • Mixed macronutrient supplement (bars)	• Never • Less than once per month • Once per month • A few times per month • Once per week • A few times per week • Daily
What is your reason for consuming (sports food)? • Sports gels • Sports confectionery • Sports bars • Sports drinks • Electrolyte supplement • Isolated protein supplement • Mixed macronutrient supplement (liquid meals) • Mixed macronutrient supplement (powders) • Mixed macronutrient supplement (bars)	• I don’t consume (sports food) • Improved performance, endurance or muscle strength, recovery • Convenience in carrying during performance • Convenience in being ready to go • Reduced risk of food spoilage on the go • Reduced stomach cramps, nausea and/or diarrhoea • Reasonable price • Less risk of banned substances • Accommodate reduced/increased energy budgets or macronutrient targets • Taste • Other
What is your reason for NOT consuming (sports food)? Please select all that apply. • Sports gels • Sports confectionery • Sports bars • Sports drinks • Electrolyte supplement • Isolated protein supplement • Mixed macronutrient supplement (liquid meals) • Mixed macronutrient supplement (powders) • Mixed macronutrient supplement (bars)	• N/A; I do consume (sports food) • Does not improve performance, endurance or muscle strength, recovery • Not convenient to carry during performance • Not convenient in being ready to go • Does not reduce risk of food spoilage on the go • Does not reduce stomach cramps, nausea and/or diarrhoea • Not a reasonable price • Risk of banned substances • Does not accommodate reduced/increased energy budgets or macronutrient targets • Taste • Other
If you have used everyday foods in place of sports foods, please provide details of what foods you have used. For example, instead of a sports bar, you might have used bread with jam or honey. • Sports gels • Sports confectionery • Sports bars • Sports drinks • Electrolyte supplement • Isolated protein supplement • Mixed macronutrient supplement (liquid meals) • Mixed macronutrient supplement (powders) • Mixed macronutrient supplement (bars)	(Free-text)
When you have used everyday foods instead of sports foods, have you found: • Performance, endurance or muscle strength, recovery • Convenience in carrying during performance • Convenience in being ready to go • Risk of food spoilage on the go • Stomach cramps, nausea and/or diarrhoea • Price • Risk of banned substances • Taste	• Much worse than sports foods • Somewhat worse than sports foods • About the same as sports foods • Somewhat better than sports foods • Much better than sports foods
Is your decision to consume sports foods (or not) influenced by:	• Advertising • Social media • Professional organisations • Peers • Friends or family • Coaches or high-performance staff • Doctors, physiotherapists or other health professionals • Personal trainers • Other
Sports foods are classified as ultra-processed foods. Ultra-processed foods have been linked to an increased risk of chronic diseases such as heart disease and depression. Are you concerned about the health effects of consuming ultra-processed sports foods? Why or why not?	• Yes • No (Free-text)
Are there any other comments you would like to make about sports foods or the questions in this survey?	(Free-text)

### Data analysis

Responses to multi-select questions were analysed using descriptive statistics with response options presented as a percentage of total responses. Pearson’s *χ* ^2^ test was used to assess potential relationships between categorical demographic variables and consumption of sports foods, with Phi and Cramer’s V used to test the strength of association and statistical significance set at *P* < 0·05. Quantitative data analysis was performed using SPSS version 28.

Responses to free-text questions were analysed thematically by AF and confirmed through review by and discussion with EM. Initial themes were identified following familiarisation with the data. All data were then coded and categorised into themes using a framework method. The brief and distinct nature of most responses enabled quantitative presentation of free-text data (i.e. percentage of responses provided aligned with a theme). Participant responses for everyday foods consumed instead of sports foods were cleaned to remove any responses unrelated to everyday foods and entered into NVivo version 12. Food items were coded according to their level of processing based on the NOVA classification groups (unprocessed or minimally processed foods; processed culinary ingredients; processed foods; UPF; and an additional category for water)^(3)^. Assumptions made when assigning codes were that homemade products used minimally processed ingredients, oats/porridge was made with rolled oats, spreads used were commercially processed, bread was mass produced, juice was shelf-stable, and unprocessed or minimally processed foods were prepared with no additional ingredients.

### Ethics

The La Trobe University Human Ethics Committee (HEC21294) and University of South Australia Human Research Ethics Committee (204227) approved this study. All participants provided implied informed consent by clicking ‘I agree’ to proceed to the survey after reading a participant information statement.

The *Strengthening the Reporting of Observational Studies in Epidemiology Statement* was used to guide the reporting of this study^(35)^.

## Results

### Participants

One hundred seventy-eight individuals commenced the survey. One hundred forty participants progressed beyond the demographic questions and were included in the reported results.

Demographic characteristics of participants are reported in Table 3. All participants were 18–74 years old; 64 % identified as women. A small number of participants (18 %) competed at a national or international level; the majority competed in individual sports (64 %) and trained 5–9 h per week (49 %). Special diets were reported by 35 % of participants. Forms of vegetarian diets, including vegan and flexitarian, were reported by 22 % of participants.

**Table 3.** Demographic characteristics of participants

	*n*	%
Age		
18–24 years	24	17·1
25–34 years	34	24·3
35–44 years	38	27·1
45–54 years	26	18·6
55–64 years	16	11·4
65–74 years	2	1·4
Gender		
Woman	89	63·6
Man	50	35·7
Non-binary	1	0·7
Annual household income (AUD)		
$0–$49 999	8	5·7
$50 000–$99 999	26	18·6
$100 000–$149 999	34	24·2
$150 000–$199 999	35	25·0
$200 000+	18	12·9
Undisclosed	19	13·6
Type of sport		
Team sport	22	15·7
Individual sport	78	55·7
Both team and individual sports	11	7·9
Non-competitive exercise only	29	20·7
Highest level of competition		
Recreational	55	39·3
Local or regional	52	37·1
State	11	7·9
National	14	10·0
International	9	6·4
Weekly hours training or competing		
0–4 h	15	10·7
5–9 h	69	49·3
10–14 h	34	24
15–19 h	13	17·1
20+ h	9	6·4
Special diets*		
No special diet	98	70·0
Flexitarian	15	10·7
Vegetarian	10	7·1
Vegan	4	2·9
Low fat	2	1·4
Low carbohydrate	6	4·3
Ketogenic	1	0·7
Gluten free	4	2·9
Low FODMAP	3	2·1
Other	8	5·7
	1 – Mediterranean	
	1 – Whole food, plant based, high protein	
	1 – High protein, clean foods	
	1 – Weekday vegan	
	1 – Lowish FODMAP but not strictly	
	1 – Pescatarian	
	1 – No dairy or nuts (allergy)	
	1 – Lactose free	

* Multiple responses permitted.

### Sports food consumption

**Fig. 1.**
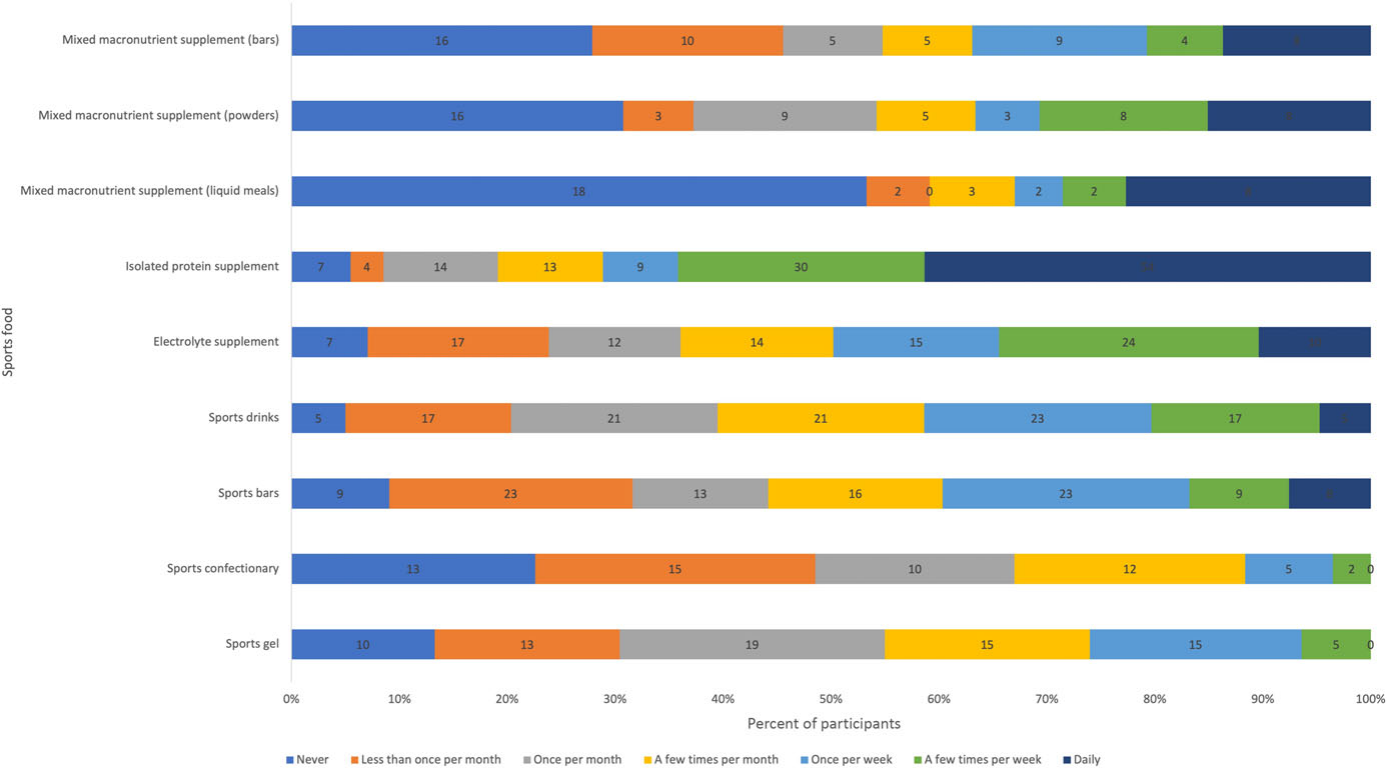
Frequency of consumption of sports foods over the past 12 months.

Nearly all participants (95 %) reported consuming any sports foods within the past 12 months (Fig. 1). Sports drinks were the most consumed sports food, reported by 73 % of participants. Liquid meals were the least consumed sports food, reported by 9 % of participants. Protein supplements were the most likely sports food to be consumed frequently, with 40 % of participants reporting consumption at least once per week (15 % daily, 21 % a few times per week, 4 % once per week).

Individual sport athletes were most likely to consume gels (χ^2^(1) = 23·087, V = 0·406, *P* < 0·001) and sports drinks (χ^2^(1) = 3·915, V = 0·167, *P* = 0·048). Athletes competing at state, national, and international levels were more likely to consume sports drinks than recreational or local athletes (85% v. 70%), but this was not statistically significant (χ^2^(1) = 3·140, V = 0·150, *P* = 0·076). Athletes participating in sport more than 10 h per week were more likely to consume sports bars (χ^2^(1) = 4·715, V = 0·184, *P* = 0·030), sports drinks (χ^2^(1) = 7·802, V = 0·236, *P* = 0·005) and electrolyte supplements (χ^2^(1) = 5·699, V = 0·202, *P* = 0·017). There were no statistically significant differences in consumption by age, gender, income or presence of a special diet.

### Motivations for sports food consumption

**Fig. 2.**
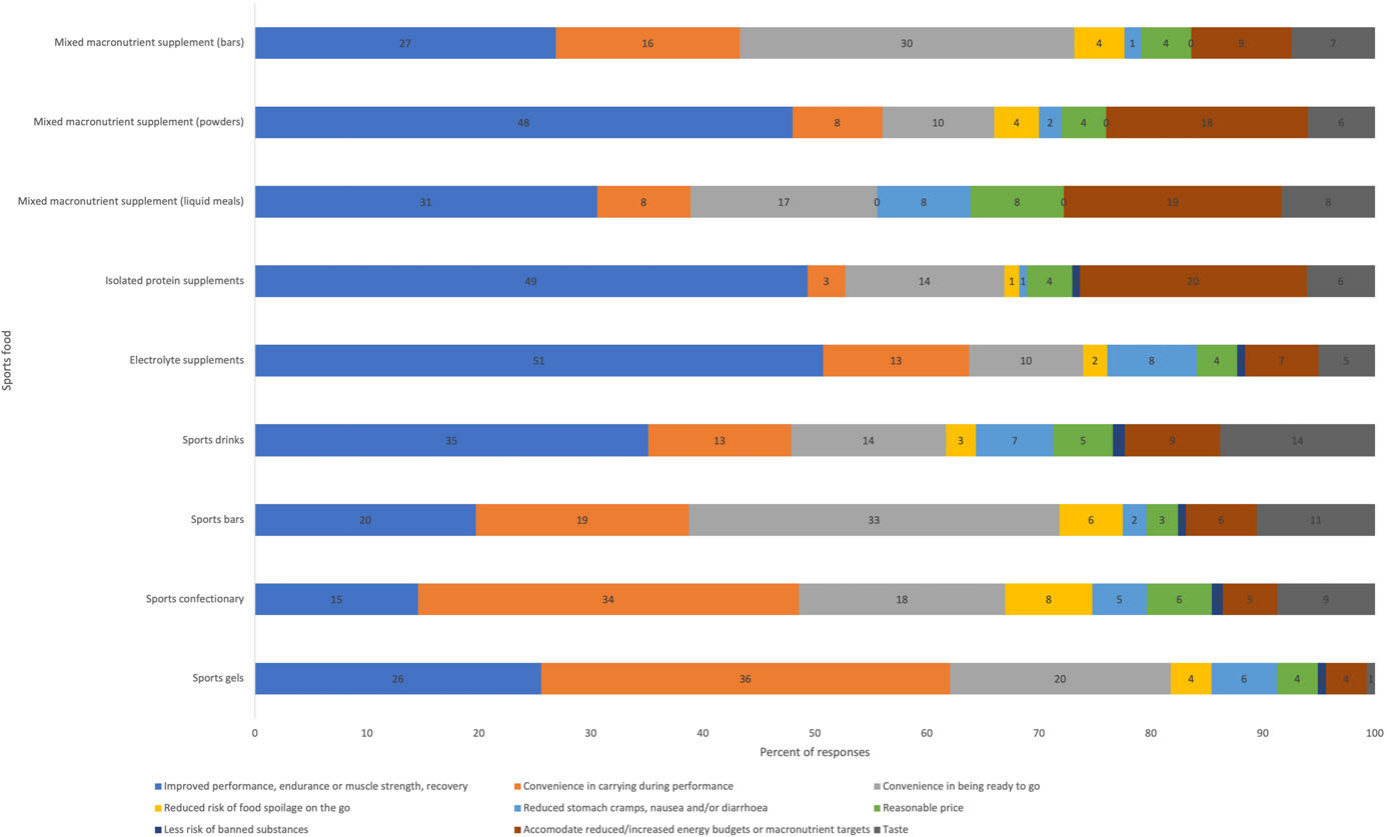
Reasons for choosing to consume sports foods.

**Fig. 3.**
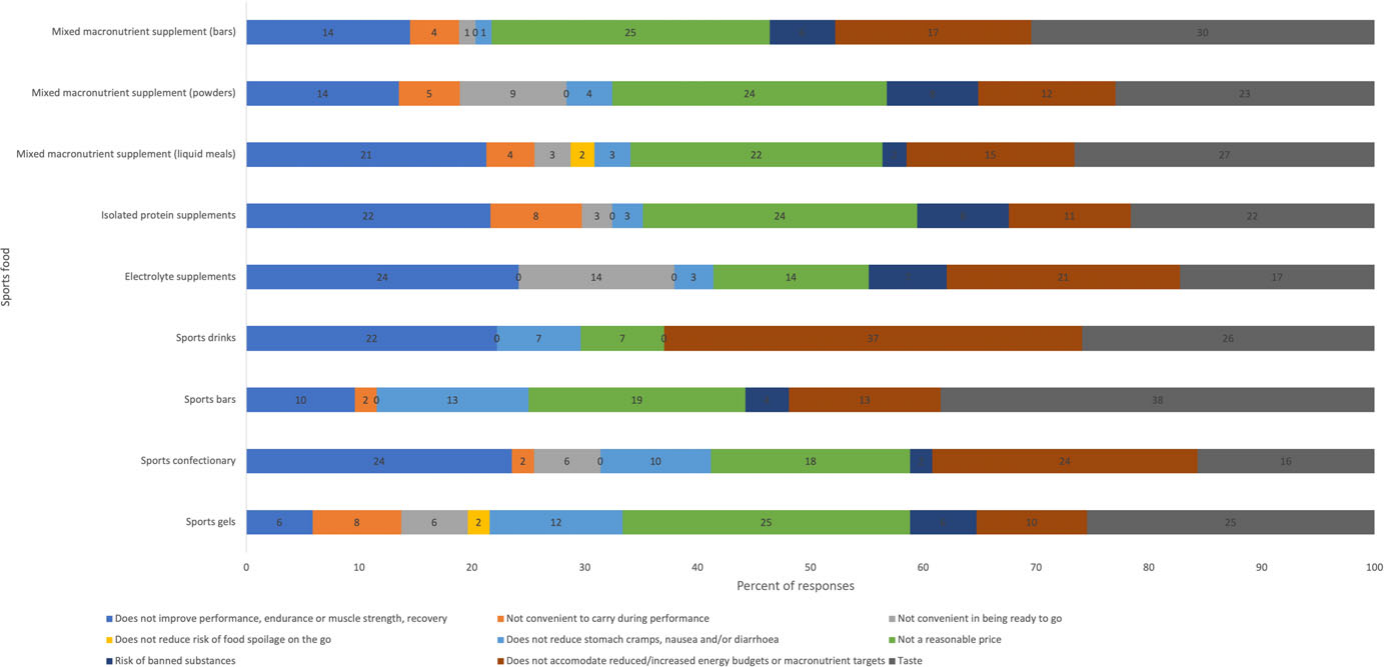
Reasons for choosing not to consume sports foods.

Performance and convenience were the most reported reasons for choosing sports foods (Fig. 2). Cost and taste were the most reported reasons for not choosing to consume sports foods, with considerations for energy/macronutrient targets or lack of performance benefit also frequently selected (Fig. 3). Relative to sports foods, participants reported that everyday foods were less convenient to prepare and to carry during performance and presented a greater risk of spoilage (Fig. 3). However, they reported everyday foods to be more affordable, taste better, present less risk of banned substances and be marginally better at supporting performance/recovery and managing gastrointestinal comfort. Peers (40 %) and coaches/high-performance staff (38 %) were the most frequently reported influencers of decisions regarding sports food consumption.

**Fig. 4.**
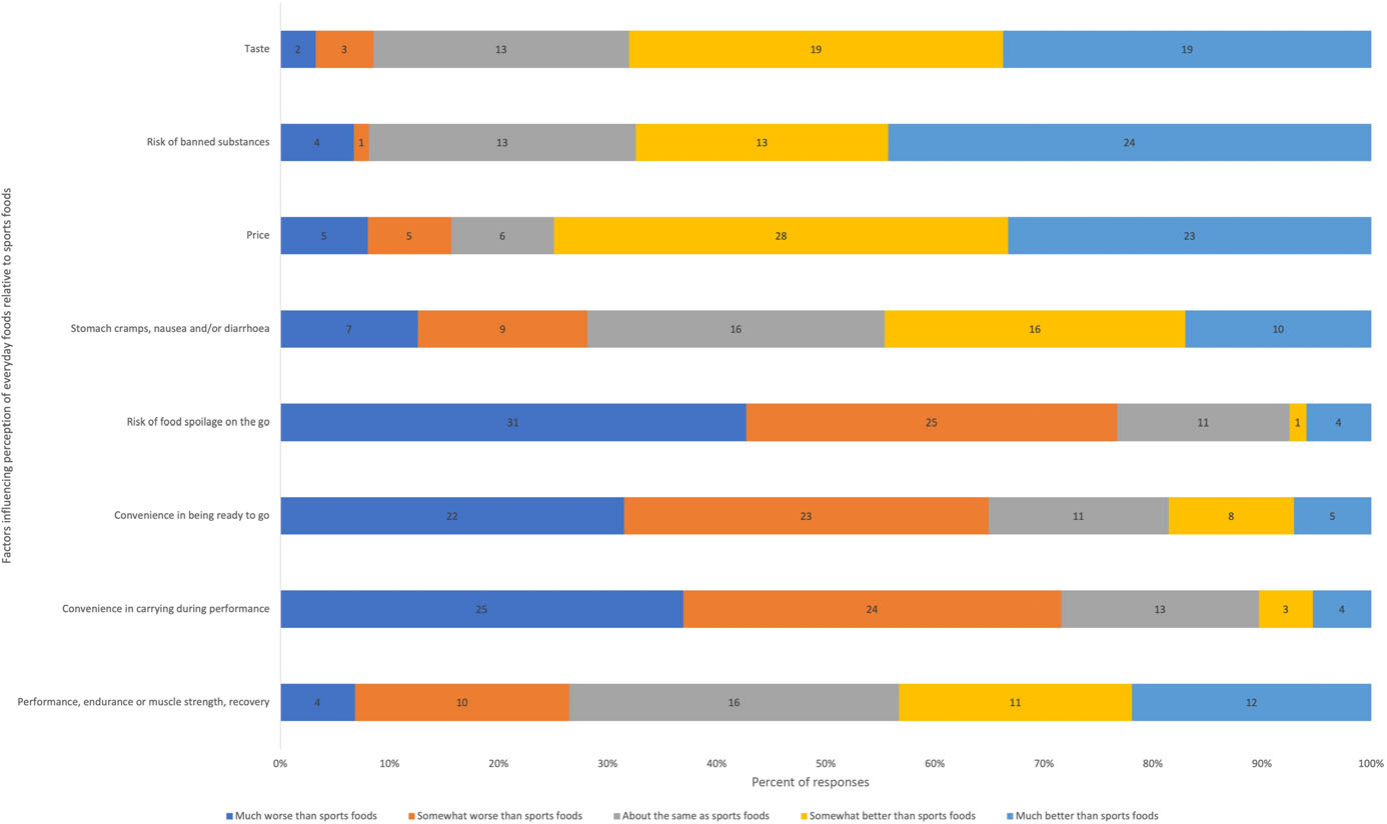
Perceptions of everyday foods relative to sports foods.

### Everyday food alternatives for sports foods

Participants described a range of foods and beverages used in place of sports foods. More than half (54 %) of the items listed by participants were UPF, such as lollies and muesli bars. Processed foods (processed foods and processed culinary ingredients), such as smoothies, whole foods (unprocessed or minimally processed foods), such as fruit, and water were listed less commonly (18, 19 and 9 %, respectively).

### Concern about ultra-processed foods

Approximately half (51 %) of participants reported concern about the health effects of consuming ultra-processed sports foods. They described concerns that these foods were not natural (33 %) or negatively impacted health (30 %). Some had specific concerns related to sugar content (9 %) or impact on gut health (15 %). Nearly two-thirds (61 %) of those not concerned about ultra-processed sports food consumption explained this was because they only used sports foods occasionally, in small amounts, or exclusively for training and performance. Others reported that the benefits related to performance or convenience outweighed the risks. Sixteen percent of those unconcerned reported they were unaware of UPF and its potential impact on health. Concern regarding consumption of ultra-processed sports foods was not related to age, gender, type of sport, level of competition or weekly hours participating in sport.

## Discussion

### Sports food consumption

In this study, almost all participants reported consuming commercial sports foods that are UPF. No other studies have considered UPF consumption specifically in athletes. In a study assessing sources of carbohydrate used in training by German endurance runners, cyclists and triathletes (*n* 1081), 87 % of athletes reported using commercial sports nutrition products (which are UPF) exclusively or combined with everyday foods to fuel their training sessions^(37)^. Most studies do not distinguish between sports foods and other sports supplements but do suggest high intakes of sports supplements among athletes^(21)^. Many (87 %) Australian athletes attending state-based sports institutes consumed sports supplements with sports foods more frequently reported than medical or performance supplements^(30)^.

Participants in our study consumed sports drinks more commonly than any other sports foods, as have athletes in other studies assessing sports food intake^(30,37)^. The study of German endurance athletes only assessed intake of carbohydrate-rich foods, so their intake of other sports foods, such as protein supplements, is unknown. Protein supplements were consumed more frequently than other sports foods in our study. Few studies have explored frequency of sports food intake (i.e. how often athletes consume sports foods). However, Italian gym users also reported frequent consumption of protein supplements, predominantly in the form of whey protein shakes^(38)^.

Participants competing at higher levels and training for longer durations were most likely to consume sports foods in our study. This suggests athletes may be using sports foods as intended – to provide nutrients and fluid required when needs are unable to be met by everyday foods^(20,21)^. For example, water is recommended as the beverage of choice for exercise up to 60–90 min with glucose/electrolyte solutions, often in the form of sports drinks, for longer bouts^(25)^. Contrary to our findings, intakes of Australian athletes attending state-based sports institutes did not differ across performance levels^(30)^. This may be related to the relatively elite status of athletes in that study compared with our more heterogeneous study population.

### Motivation for sports food consumption

Participants in our study stated they consumed sports foods for performance and convenience. Performance may be a particularly important concern for those competing at higher levels and may explain the previously noted higher sports supplement intake among higher-calibre athletes^(23)^. Performance was the strongest influence of food choice among athletes attending the 2018 Commonwealth Games and 2017 Universiade competitions^(39)^, while convenience was the primary reason why German endurance athletes selected sports foods over everyday foods^(37)^.

Participants in our study cited taste and cost as reasons for not consuming sports foods. Taste, as an element of sensory appeal, was ranked second behind performance as a factor influencing food choice of 2018 Commonwealth Games and 2017 Universiade athletes in Thurect and Pelly’s study^(39)^ but was considered by only 15 % of German endurance athletes when choosing carbohydrate-rich foods for training sessions^(37)^. Reinhard and Galloway suggest this discrepancy may be related to Birkenhead and Slater’s assertion that the importance of taste varies by eating occasion^(40)^. So, taste preferences for everyday foods may not override performance-driven motivations for sports foods. While cost was cited as a reason for not consuming sports foods in our study, the influence of cost on athlete food choice in other studies seems to vary depending on the cohort and the context^(39–41)^. Athletes with limited disposable income who are responsible for purchasing and providing their own food are most likely to be influenced by cost, and we did not assess these contextual factors. Regardless, as many everyday foods can also be cost-prohibitive, athletes and their support teams may need suggestions for low-cost alternatives to commercial sports foods.

Athletes are commonly concerned about doping when making food choices^(30,39)^. Doping is an important consideration, particularly for athletes competing at levels of competition subject to World Anti-Doping Authority regulations^(42)^. Participants in our study believed that everyday foods presented a lower risk of banned substances than commercial sports foods. However, some protein-fortified everyday foods, such as those prepared by cafés and other food outlets, can present a doping risk for athletes, with batch-tested commercially prepared protein-fortified foods a safer option for athletes looking for convenient high-protein options^(43)^. Therefore, athletes and their support teams may need further education about doping risks of everyday and sports foods.

In our study, participants reported that peers and coaches/high-performance staff influenced their decisions about sports foods. An earlier review found that coaches, trainers, friends and family were the most commonly used sources of dietary supplement information for athletes^(44)^. In other Australian studies, athletes have reported coaches, sports scientists/physicians, dietitians/nutritionists, peers and the internet as sources of nutrition and sports supplement information^(12,29,30,45,46)^. While it is important to understand who influences athletes’ food choices, few (< 10 %) German endurance athletes were influenced by peers, nutritionists or coaches to consume everyday foods over commercially prepared sports foods^(37)^. Given that food choice, particularly for individual endurance sport athletes, may be influenced more by their personal views than by others, sporting organisations should focus education efforts on athletes themselves.

### Everyday food alternatives for sports foods

In this study, more than half of the everyday food items consumed by participants in place of commercial sports foods were UPF. So, athletes may need education on the risk of UPF foods and support to identify non-UPF everyday food options. Furthermore, convenient access to safe and affordable minimally processed options may support athletes to make these changes in practice. Education and support for training and competition venues to provide non-UPF options may lead to enhanced access to these foods where they are most convenient to athletes. Other research has demonstrated that healthy canteen displays at sporting association trade shows can support organisations to plan and implement changes in their canteens^(47)^.

### Concern about ultra-processed foods

Approximately half of the participants in our study were concerned about the health effects of consuming UPF. Participants unconcerned about the health impacts of UPF reported that use was only occasional, and that performance and convenience benefits outweighed health risks. Education for athletes and their support teams should highlight that negative health consequences of UPF exist regardless of overall diet quality^(11)^ and that any UPF consumption may harm their overall health. They can then make informed decisions about whether to consume ultra-processed sports foods as part of their performance plan.

### Strengths and limitations

Our study explored the views of a broad range of athletes across Australia. The sample included a broad distribution of responses from women and men and athletes across diverse sport types and participation levels. Consequently, the results are likely to be generalisable to Australian athletes. Since each country has specific guidance on sports supplements, it is important to conduct supplement-related research within the context of the host country.

We based the survey on a previously validated survey and piloted the adapted version to assess content and face validity before use. The survey forced responses and provided ‘other’ options and free-text boxes throughout the survey to ensure the most relevant options were covered. A combination of question types, including free-text, enabled investigation into both sports food practices and reasons for choices made by athletes. However, we evaluated only the frequency of sports food consumption, not actual amounts consumed or intake as a proportion of total energy intake. Further, we evaluated only sports food intake, not overall diet. As we conducted this study before publication of the Participant Classification Framework^(48)^, participation categories are similar but not fully aligned with the six-stage framework. The five categories presented align with Tiers 1–4, and it is possible, though unlikely, that some athletes in this study may classify as Tier 5 (World Class).

Gibney *et al*.^(49)^ suggest that classification of foods into NOVA categories can be problematic and Astrup and Monteiro^(50)^ note the lack of consistency in classification of foods by researchers in other studies. So, while in our experience the classification of foods consumed in place of commercial sports foods was straightforward and there was agreement between researchers, it is possible that others may have interpreted and classified the foods differently. To distinguish between categories, we made assumptions for some foods and we have noted these in our methods.

Finally, several publications dispute the NOVA classification system and potential health concerns associated with UPF^(50,51)^. These focus on the idea that the NOVA classification does not add to public health messaging beyond a nutrient or dietary pattern approach. Nonetheless, athletes should consider the impact of added harmful compounds and lost protective elements on health and performance, even when the nutrient composition of a manufactured food product aligns with their sports nutrition plan and dietary targets. At this stage, there is no evidence to demonstrate whether the benefits of exercise can mitigate the adverse health effects of consuming UPF.

### Recommendations for future research

Regulations guiding product content, labelling and claims can help athletes and their teams make informed decisions about sports supplements including sports foods^(52)^. However, they do not address the potential health risk of consuming UPF. Given Dicken and Batterham’s^(11)^ study demonstrating that healthful dietary patterns do not mitigate the chronic health risks of UPF, concerns and unanswered questions remain regarding sports food recommendations for athletes. Future studies are needed to identify whether there is an association between sports food consumption and the development of chronic disease. Further investigations should explore the type, frequency, amount and duration of sports food consumption in various athletic populations and whether any risks are offset or increased by exercise type, frequency and duration. At a practical level, sporting organisations should trial providing education about and access to safe, affordable, convenient, minimally processed food options for athletes and their support teams. Finally, addressing UPF as a strategy to support more sustainable diets for athletes can be explored^(53)^.

### Conclusion

Almost all participants in this study consumed ultra-processed commercial sports foods as convenient options to support performance. Half of the participants were concerned about the potential adverse health effects of consuming UPF. Existing literature suggests that an overall healthy dietary pattern does not mitigate the impact of UPF on health, and more research is needed to understand the impact of exercise. Athletes may consider reducing UPF intake to lower their risk of developing diet-related disease. Level of processing may be used as a simple guide for athletes to adopt health-promoting dietary behaviours that align with their performance nutrition goals. Athletes and their support teams need education and access to safe, affordable, convenient, minimally processed alternatives to commercial sports foods.
